# Spontaneous Pneumothorax in an Elderly Patient With Coronavirus Disease (COVID-19) Pneumonia

**DOI:** 10.31486/toj.20.0072

**Published:** 2020

**Authors:** Tyler Rehman, Greta Josephson, Moutaz Sunbuli, Amar R. Chadaga

**Affiliations:** ^1^ Internal Medicine Residency, University of Illinois at Chicago/Advocate Christ Medical Center, Oak Lawn, IL; ^2^ Department of Pulmonary and Critical Care Medicine, Advocate Christ Medical Center, Oak Lawn, IL; ^3^ Department of Internal Medicine, Advocate Christ Medical Center, Oak Lawn, IL

**Keywords:** *COVID-19*, *pneumothorax*, *SARS-CoV-2*

## Abstract

**Background:** The relationship between the 2019 novel coronavirus (COVID-19) and pneumothorax is not yet established. As of June 2020, few cases of nonintubated patients developing pneumothorax had been documented.

**Case Report:** We present the case of an elderly patient with COVID-19 pneumonia that resulted in a prolonged hospital course because of pneumothorax complication. The patient did not develop severe symptoms and did not require intubation.

**Conclusion:** This case report should aid clinicians assessing patients with COVID-19 pneumonia.

## INTRODUCTION

Pneumothorax can be subdivided into 3 categories: spontaneous, traumatic, and iatrogenic.^1^ Spontaneous pneumothorax is an abnormal accumulation of air in the space between the lungs and the pleural space that can result in the partial or complete collapse of a lung. Spontaneous pneumothorax is usually attributed to a bleb rupture.^2^ The relationship between severe acute respiratory syndrome coronavirus 2 (SARS-CoV-2, the 2019 novel coronavirus [COVID-19]) and pneumothorax is not yet established. To our knowledge as of June 2020, we have identified few other documented cases of pneumothorax seemingly related to COVID-19 in nonintubated patients.

## CASE REPORT

An 83-year-old female presented to the emergency department with 3 weeks of worsening exertional shortness of breath and orthopnea. The patient's medical history included chronic kidney disease stage 3B, coronary artery disease, and systolic heart failure. During her presentation, she was tachypneic to 22 respirations per minute, and pulse oxygenation was 97% on 4 L nasal cannula. Initial chest radiograph revealed bilateral scattered fine interstitial opacities, right greater than left, and laboratory examination was significant for N-terminal prohormone of brain natriuretic peptide of 33,602 pg/mL, raising suspicion that the patient was hypervolemic. The patient's other notable laboratory findings were elevated creatinine of 1.95 mg/dL (stable from previous testing), troponin of 0.52 ng/mL, and lactate dehydrogenase of 432 u/L (Table).

**Table.** Initial Laboratory Results

Test	Result	Normal Range
Creatinine, mg/dL	1.95	0.51-0.95
White blood cells, 10^9^/L	5.4	4.2-11.0
Troponin, ng/mL	0.52	<0.05
N-terminal prohormone of brain natriuretic peptide, pg/mL	33,602	<451
Aspartate aminotransferase, u/L	52	<38
Alanine aminotransferase, u/L	32	<64
Lactate dehydrogenase, u/L	432	82-240

The patient was admitted for possible congestive heart failure exacerbation and treated with intravenous furosemide 40 mg twice daily. She was concomitantly treated empirically for community-acquired pneumonia with ceftriaxone 2 g daily for 5 days and azithromycin 500 mg daily for 5 days. Echocardiogram revealed reduced ejection fraction of 40% to 45% (stable from previous examinations). The patient was classified as a person under investigation, and shortly after admission, she tested positive for SARS-CoV-2, which led to the diagnosis of COVID-19 pneumonia. The patient's troponin was trended and remained stable, and her electrocardiogram did not show evidence of acute ischemic changes. The cardiology team attributed her elevated troponin to type 2 myocardial infarction in the setting of viral pneumonia.

On the patient's third day of hospitalization, her oxygen requirements increased to 6 L nasal cannula, and she developed significant oxygen desaturations with exertion. Repeat chest radiograph was ordered because of her worsening hypoxemia and revealed a 30% to 40% right-sided pneumothorax (Figure). The patient initially declined chest tube thoracostomy and opted for conservative management. Her oxygen requirements increased to 15 L nonrebreather mask on day 5 of hospitalization, and repeat chest radiograph demonstrated enlargement of the pneumothorax. The patient agreed to proceed with chest tube placement at that time. After chest tube placement, the patient's oxygen requirements began to improve. Because of the patient's clinical improvement, the pulmonary team did not recommend chest computed tomography (CT).

**Figure.**
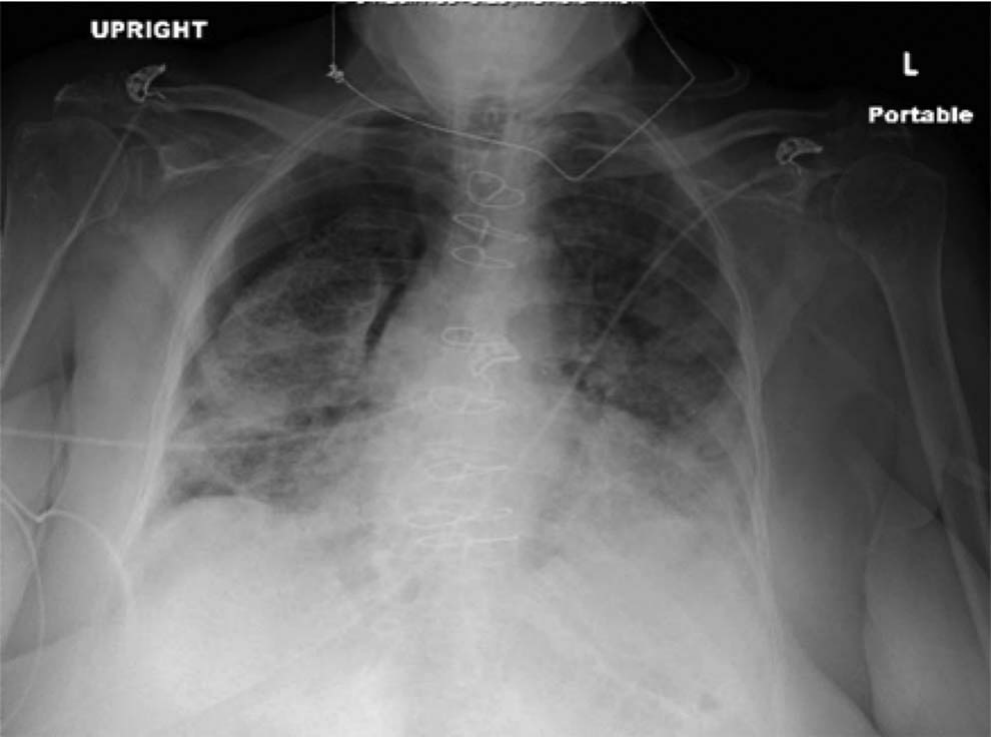
**Chest radiograph revealed a right-sided 30% to 40% pneumothorax.**

On day 15 of hospitalization, the patient's chest tube was unintentionally partially dislodged. Chest radiograph at that time showed a small persistent apical pneumothorax; however, no leak was reported. The pulmonary team adjusted the chest tube and connected it to water seal. Repeat chest radiograph showed stable pneumothorax, and the chest tube was removed on day 16 of hospitalization. The patient was discharged home in stable condition on day 18 of hospitalization on 1 L nasal cannula.

## DISCUSSION

Spontaneous pneumothorax can be subdivided into primary or secondary. Primary spontaneous pneumothorax (PSP) occurs in patients without known lung disease. Secondary spontaneous pneumothorax (SSP) occurrences are attributed to an underlying lung disease. SSP usually occurs in older patients and can often be linked to genetic causes.^3^ Many factors can increase the risk for spontaneous pneumothorax such as age, smoking, and sex. A study done in the United Kingdom reported that spontaneous pneumothorax appeared to present in a bimodal distribution (age 15 to 34 years and age >55 years). This distribution appeared to correlate with PSP being more common in the younger age group (15 to 34 years) and SSP being more common in the older age group (>55 years).^4^

Although COVID-19 data are still being collected, other viral pneumonias have been associated with pneumothorax. In a study published in 2000, 1.2% of patients with pneumonia attributed to human immunodeficiency virus (HIV) were found to have pneumothorax.^5^ The patients with HIV pneumonias were commonly diagnosed with other superimposed infections.

Spontaneous pneumothorax associated with COVID-19 in nonintubated patients was not a commonly reported complication in June 2020. One case documented in South Korea involved a young, otherwise healthy male whose severe cough was thought to be a potential cause for the pneumothorax.^1^ Another case involved a 50-year-old male who developed a pneumothorax as a late sequela of COVID-19.^6^ The patient reportedly was recovering from his infection at home with supplemental oxygen when the pneumothorax occurred.

Pneumothoraces in patients with COVID-19 were documented in 2 published reports from Wuhan, China; pneumothorax was identified in 1 of 92 COVID-19–positive patients and 1 of 99 COVID-19–positive patients, respectively.^7,8^ A systematic review of CT imaging of 919 COVID-19–positive patients described pneumothorax as an uncommon complication but one that was observed with disease progression.^9^

Our patient was not intubated at any point during her hospitalization, and she did not require additional noninvasive breathing assistance beyond the nonrebreather mask. She did not have a severe cough, hemoptysis, or significant tobacco use history. She denied any known medical history of lung or respiratory problems. The patient was diagnosed with SSP because of her viral pneumonia. Her viral pneumonia symptoms were relatively mild at presentation, so chest CT was not ordered on arrival. She did not exhibit significant cough or respiratory distress, and her hypoxia was initially attributed to pneumonia and heart failure. CT studies can be helpful in providing detailed information regarding lung structure and disease progression as described in South Korean and Chinese reports.^1,10^ When considering other viral pneumonias as reported by Afessa, the superimposed bacterial infection could have potentially increased the patient's risk for pneumothorax.^5^ Our patient empirically completed a course of treatment for community-acquired pneumonia with ceftriaxone and azithromycin.

As the patient's oxygen requirements increased, her clinical picture did not appear to be consistent with pneumo-thorax. Rather, her symptoms were initially attributed to the progression of her viral pneumonia.

## CONCLUSION

This case underlines the importance of considering repeat examinations such as chest radiographs or CT imaging for thorough evaluation of COVID-19–positive patients. The prevalence of pneumothorax in COVID-19–positive patients is unknown. Until more data are available, a reasonable diagnostic course is to consider ruling out pneumothorax when oxygen requirements worsen in patients diagnosed with COVID-19.

## Notes

This article meets the Accreditation Council for Graduate Medical Education and the American Board of Medical Specialties Maintenance of Certification competencies for Patient Care and Medical Knowledge.
